# Supplementary material for: An Efficient and Rapid Protocol for Somatic Shoot Organogenesis from Juvenile Hypocotyl-Derived Callus of Castor Bean cv. Zanzibar Green
Source: BioTech (Basel). 2024 Jul 4;13(3):25. doi: 10.3390/biotech13030025 (PMC11270351; doi:10.3390/biotech13030025)
Supplement: Supplementary file 1 [file biotech-13-00025-s001.zip › biotech-2986051-supplementary.pdf]

An Efficient and Rapid Protocol for Somatic Shoot Organogenesis from Juvenile Hypocotyl-Derived Callus of Castor Bean cv. Zanzibar Green

Danaya V. Demidenko, Nataliya V. Varlamova, Taisiya M. Soboleva, Aleksandra V. Shitikova and Marat R. Khaliluev

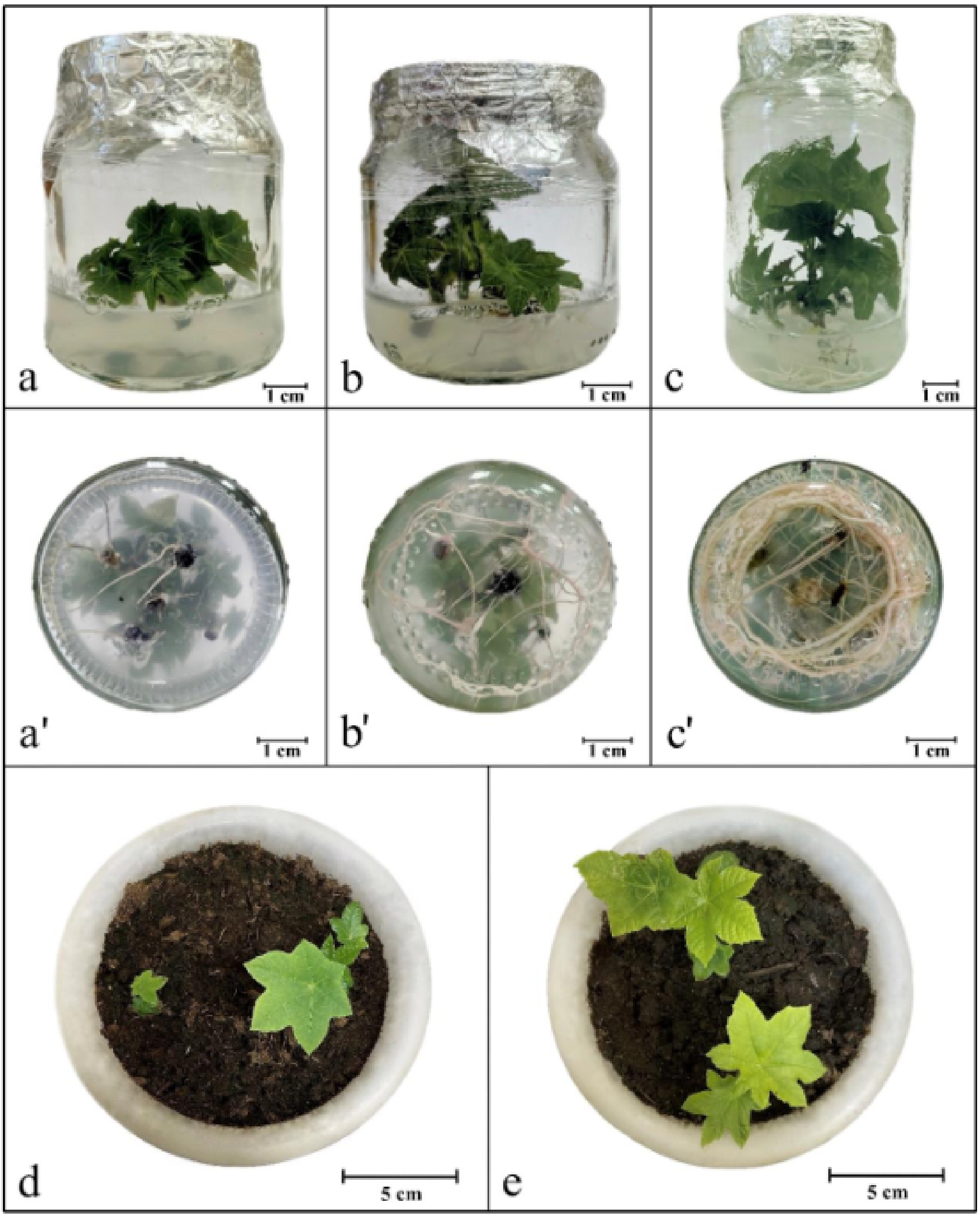

Figure S1. In vitro root induction from regenerants on ½ MS medium without PGRs after 7 (a, a'),.21 (b, b') and 56 (c, c') days of culture, as well as plantlets adaptation to ex vitro conditions after 3(d) and 14 (e) days of cultivation in soil substrate.
